# Recombinant inbred lines derived from wide crosses in *Pisum*

**DOI:** 10.1038/s41598-023-47329-9

**Published:** 2023-11-21

**Authors:** N. Ellis, J. Hofer, E. Sizer-Coverdale, D. Lloyd, G. Aubert, J. Kreplak, J. Burstin, J. Cheema, M. Bal, Y. Chen, S. Deng, R. H. M. Wouters, B. Steuernagel, N. Chayut, C. Domoney

**Affiliations:** 1 John Innes Centre, Norwich Research Park, Colney Lane, Norwich, NR4 7UH UK; 2 Institute of Biological, Environmental and Rural Sciences, Aberystwyth University, Plas Gogerddan, Aberystwyth, SY23 3EB UK; 3 Agroécologie, INRAE, Institut Agro, Univ. Bourgogne, Univ. Bourgogne Franche-Comté, 21000 Dijon, France; 4 Present Address: Germinal Horizon, Institute of Biological, Environmental and Rural Sciences, Aberystwyth University, Plas Gogerddan, Aberystwyth, SY23 3EB UK

**Keywords:** Agricultural genetics, Genetic linkage study, Plant genetics

## Abstract

Genomic resources are becoming available for *Pisum* but to link these to phenotypic diversity requires well marked populations segregating for relevant traits. Here we describe two such resources. Two recombinant inbred populations, derived from wide crosses in *Pisum* are described. One high resolution mapping population involves cv Caméor, for which the first pea whole genome assembly was obtained, crossed to JI0281, a basally divergent *P. sativum sativum* landrace from Ethiopia. The other is an inter sub-specific cross between *P. s. sativum* and the independently domesticated *P. s. abyssinicum*. The corresponding genetic maps provide information on chromosome level sequence assemblies and identify structural differences between the genomes of these two *Pisum* subspecies. In order to visualise chromosomal translocations that distinguish the mapping parents, we created a simplified version of Threadmapper to optimise it for interactive 3-dimensional display of multiple linkage groups. The genetic mapping of traits affecting seed coat roughness and colour, plant height, axil ring pigmentation, leaflet number and leaflet indentation enabled the definition of their corresponding genomic regions. The consequence of structural rearrangement for trait analysis is illustrated by leaf serration. These analyses pave the way for identification of the underlying genes and illustrate the utility of these publicly available resources. Segregating inbred populations derived from wide crosses in *Pisum*, together with the associated marker data, are made publicly available for trait dissection. Genetic analysis of these populations is informative about chromosome scale assemblies, structural diversity in the pea genome and has been useful for the fine mapping of several discrete and quantitative traits.

## Introduction

Recombinant inbred lines (RILs) were first developed for mouse genetics^1^ but are widely used in plant genetics where self-fertilization makes their development relatively straightforward. RILs capture genetic variation in a stable way. As inbred lines they are amenable to multiple investigations, such as replicated measurement or the accumulation of data over time. There are two disadvantages to RILs: they do not capture information about dominance unless this is recorded in early generations and they segregate only for the alleles that distinguish the two parents. The latter disadvantage is overcome by linkage disequilibrium mapping methods such as Multi-parent Advanced Generation Inter-Cross (MAGIC) populations^2^, Nested Association Mapping (NAM)^3^ which, together with Genome-Wide Association Studies (GWAS), enable analyses of diverse populations^4^ and can capture the contribution of multiple alleles. Nevertheless, sufficiently large RIL populations can provide a high degree of resolution in genetic mapping and, when the parents are sufficiently divergent, RILs can capture many bi-allelic differences.

Here we present a preliminary analysis of two RIL populations derived from two wide crosses in *Pisum*. A wide cross within *P. sativum sativum* is represented by RILs derived from the cross between cv Caméor (a French field pea variety, also designated JI3253) and JI0281 (a *P. s. sativum* accession from Ethiopia, designated ‘*P. sativum* landrace DCG0248’ in Kreplak et al.^5^). The second is an inter-specific or inter-subspecific cross. One parent of this second wide cross is JI2202 (designated ‘*P. sativum abyssinicum*_Landrace_DCG0563 by Kreplak et al.^5^) which represents a closely related group of peas that have been domesticated independently from *P. s. sativum*^6,7^. Sometimes *P. s. abyssinicum* is regarded as a distinct species rather than a subspecies of *P. sativum*. The second parent of this population, JI2822, is a genetic stock, a RIL derived from the cross between JI0015 and JI0399, which has been widely used in mutagenesis experiments (see^8^ and references therein) or for gene content analysis^9^. These RIL populations were generated to take advantage of the large genetic distance between these sequenced genomes^5^, enabling the creation of high-density genetic maps for use in verifying genome assemblies and they are also intended as a stable resource for trait analysis and gene discovery in pea. The RILs and their associated data are publicly available from the Genetic Resources Unit of the John Innes Centre (https://www.seedstor.ac.uk/search-browseaccessions.php?idCollection=16).

## Results

### JI0281 × Caméor

A recombinant inbred population derived from the cross between cv Caméor (also designated JI3253) and JI0281 (designated ‘*P. sativum* landrace DCG0248’ in Kreplak et al.^5^) was generated to take advantage of the large genetic distance between these two sequenced genomes (Kreplak et al.^5^). Caméor is a French field pea variety, and JI0281 is a *P. sativum* accession from Ethiopia. For the JI0281 × Caméor RIL population, an Axiom SNP array with 84,691 features was used to score markers segregating in the F6 and F7 generations. The F7 individuals were derived from the F6 individuals of which a subset was genotyped. The set of markers which gave useful data was slightly different for the two data sets, presumably reflecting slightly different assay conditions.

The F6 data set comprised 375 individuals and 17,936 markers. In the F7 generation 570 RILs were scored with 18,349 markers.

#### F6 and F7 RI Lines

Those RILs for which marker data exists at the F6 and F7 generation can be compared to validate the correspondence between the F6 and F7 individuals, their marker scores and the position of crossover events. This analysis showed that some RILs of the same generation share a parent at a generation later than the F1. 366 RILs were genotyped in both the F6 and F7 generations. 10 RILs were genotyped in the F6 alone and 204 were genotyped only in the F7. The relationships between the F6 and F7 individuals is given in Supplementary Table 1 and the allele call frequencies according to call category are given in Supplementary Table 2.

#### Crossovers per linkage group

**Figure 1:**
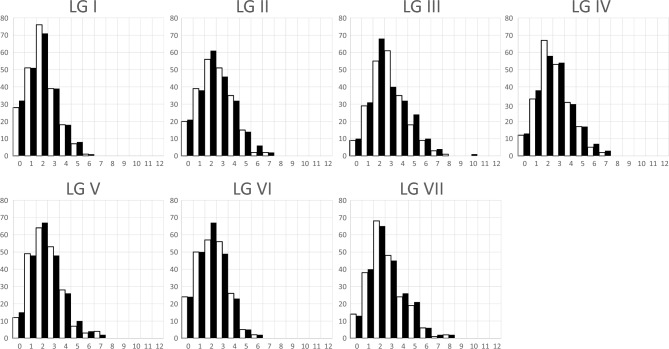
Frequency distribution of recombination events in the subset of matched RILs at F_6_ and F_7_. A change from one homozygous class to the other was counted as one recombination event and a change between homozygosity and heterozygosity was counted as ½. The sum of events corresponds to the expected crossover frequency per gamete. The x-axis is the number of recombination events and the y-axis is the number of instances. Black bars represent F6 generation, white bars represent F7 generation.

The data quality, as measured by the number of missing scores, is better for F6 than F7 RILs. In total, 14,492 segregating markers were scored in both the F6 and F7 generations and the number of missing scores per RIL were 26.47 ± 50.20, N = 357 and 243.37 ± 340.41, N = 571 (μ ± SD, N) respectively. For a subset of 240 matched F6 and F7 RILs, where there were no more than 300 missing scores (ca. 2%), a linkage map was constructed, and the frequency distribution of recombination events was calculated as shown in Table 1 and Fig. 1.

**Table 1:** Crossover number by linkage group and generation.

LG	I		II		III		IV		V		VI		VII	
	F6	F7	F6	F7	F6	F7	F6	F7	F6	F7	F6	F7	F6	F7
μ	1.91	1.90	2.44	2.44	2.87	2.81	2.56	2.57	2.33	2.26	2.15	2.06	2.55	2.58
var	1.53	1.66	2.16	2.39	2.24	2.56	1.95	2.14	1.95	1.92	1.71	1.61	2.34	2.4

The mean and variance of the number of crossovers per RIL (N = 240 for F6 and F7). The variance is given because the expectation for a Poisson distribution is that these should be equal.

The total number of recombination events in the F6 and F7 generations is very similar (4034 vs 3989.5 respectively). All recombination events between homozygous segments in the F6 are expected to be preserved in the F7. Half of the recombination events between homozygous and heterozygous segments in the F6 are expected to be retained in the F7; a quarter should segregate as non-recombinants and a quarter as recombinants between homozygous segments. New recombination events within heterozygous segments will generate additional recombination events. An example of the observed recombination events in one pair of F6 and F7 RILs is illustrated in Supplementary Fig. 1.

In the F6 and F7 approximately 8% of linkage groups were non-recombinant (129 and 139 respectively); this is as expected from a Poisson distribution of recombination events (Table 1, Fig. 1).

For 287 of the 366 pairs of corresponding F6 and F7 RILs, the fraction of differing marker scores was 0.022 ± 0.017 (μ ± SD). The expectation for an F6 is that 0.5^5^ markers will be heterozygous and, of these, half will be heterozygous in the next generation, so the expected frequency of marker differences is 0.5^6^ (0.012625). The slight excess corresponds to mis-called alleles.

#### Genetic map order and distance

Additional maps were constructed for the F6 and F7 generation data in order to compare marker order and map distances. For the F7 generation 66 RILs were not used for map construction because they had more than 500 missing allele calls. In the F6 generation the fraction of alleles called as heterozygous is 0.0356, close to the expectation of 1/32. In the F7 the fraction is 0.015, again close to expectation.

Two methods of map construction were used. The first was based on an initial order of markers according to their predicted physical position in the Caméor v1a genome assembly^5^ to obtain a graphical genotype. This identified any markers in a position inconsistent with the minimum number of recombination events. These markers were removed temporarily, and, after a scaffold map was made, they were replaced in a position that minimised the number of recombination events required according to the method of^10^. The second map construction method was a modified version of Threadmapper^11^ as described below.

### Threadmapper

The Threadmapper method of ordering genetic markers is convenient for large data sets as it quickly gives a visual representation of map quality. The method is essentially a principal component analysis of the inter-marker distances whereby points on a linkage group are located on a curve in the multidimensional space. The order of markers along this curve is the order of the markers on the genetic map and the orthogonal distance of a marker from the curve increases as marker quality decreases^11^. The method works conveniently for three-dimensional spaces, which is particularly useful for dissecting the relationship between linkage groups, especially if translocations are segregating (see later). In this analysis we simplified the approach of Cheema et al.^11^ by performing the multivariate analysis on the markers by RILs data rather than the distance matrix (markers × markers) because the markers by RILs data set is smaller, which makes computation faster. For RIL populations, the data is simply encoded as + 1 or − 1 for the two parental alleles, with missing data and heterozygotes treated as 0.

**Figure 2:**
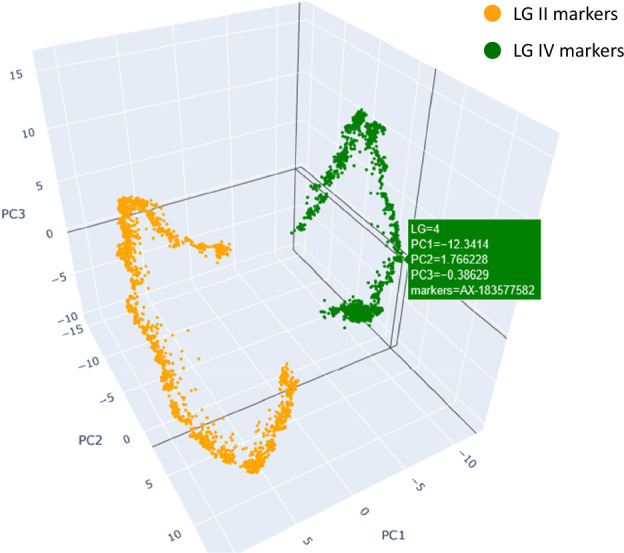
Threadmapper projection of markers on LG II and LG IV of the F7 RIL population of the JI0281 × Caméor RIL population. Markers are coloured according to their chromosomal assignment in the ZW6 genome. An interactive version is available in the supplementary file 281xCameor_F7_LG2_LG4.html; see also the whole data sets in 281xCameor_F6.html and 281xCameor_F7.html.

The F6 and F7 linkage maps generated by Threadmapper can be seen in the accompanying interactive displays, 281xCameor_F6.html and 281xCameor_F7.html, respectively. Linkage groups II and IV appear close in the latter display but are clearly distinct when they are displayed as a pairwise comparison of 281xCameor_F7_LG2_LG4 (Fig. 2).

### Map resolution and comparison to physical maps

For the set of markers scored in both the F6 and F7 generation map distances were calculated from the fraction of RILs that had recombined the parental alleles, together with the map expansion function of Haldane and Waddington^12^ and Haldane’s mapping function^13^. The map expansion function^12^ relates the fraction of inbred lines *R* that have recombined parental alleles to the recombination rate *r* per meiosis. For extensively selfed inbred lines the relationship is *r* = *R*/2(1 − *R*), so using this relationship to estimate *r* in early generation progeny underestimates *r*. When *R* (or *r*) is small, *r* ≈ *R*/2. Haldane’s mapping function^13^ *d* = − ½ln(1− 2*r*) [*d* is in Morgans, for centimorgan distances ½ is replaced by 50] provides an additive function of recombination rates, and note that for small values *r* ≈ *d*. For ca. 500 RILs the minimum (non-zero) observable value of *R* is 1/500, *r* ≈ 0.001, *d* ≈ 0.1 cM.

**Figure 3:**
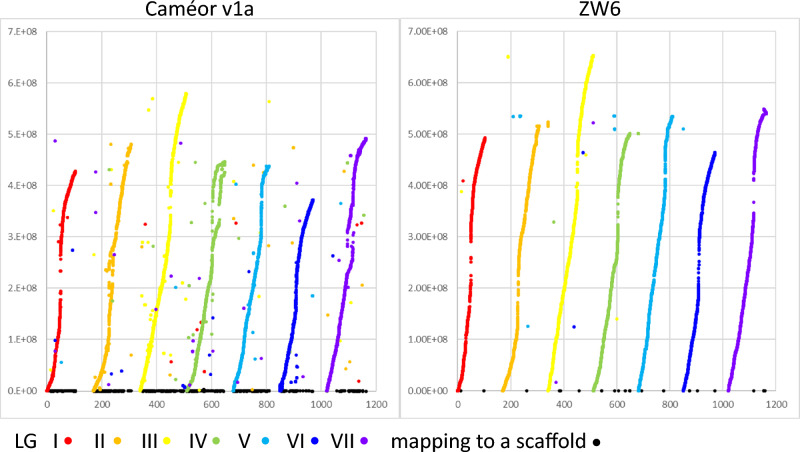
Genetic map compared to genome assemblies. The genetic map of JI0281 × Caméor (x-axis, in cM cumulatively) from the set of markers common to the F6 and F7 genotypes is compared to genome assemblies (y-axis, in nucleotides) for Caméor^5^ and ZW6^14^.

The genetic map position of these markers is compared to published genome assemblies in Fig. 3.

The average spacing between markers on the JI0281 × Caméor genetic map is 0.065 ± 0.137 (μ ± SD, N = 14,491) cM, with the maximum distance between adjacent markers being 2.078 cM. The average spacing between adjacent genetic map markers on the Caméor v1a assembly, where the assembly and map are in alignment, is 1.36 ± 11.91 Mb (μ ± SD, N = 7085). For the ZW6 assembly^14^, where the alignment is better (Fig. 3), the average spacing between adjacent genetic map markers is 0.26 ± 0.94 Mb (μ ± SD, N = 13,648).

### Comparing the F6 and F7 maps

The genetic maps constructed for the individual F6 and F7 generations differed both in the number of lines and the number of markers which were scored and, as noted above, the data quality for the F6 generation was better than for the F7. The F6 had ca. 0.14% missing data and the F7 had ca. 1.3% missing data. These maps are compared to the common F6 and F7 map discussed above, as shown in Supplementary Fig. 2. The excess length of the F7 map is distributed throughout the linkage groups, but the difference is less pronounced in linkage groups I, IV, VI and VII. Presumably this reflects the distribution of unreliable marker scores. This effect is partly remedied in the map derived from both populations because mis-scores are unique to one or other generation and furthermore because each marker and line is replicated unreliable scores can be seen in the graphical genotypes (see Supplementary data file 281xCameor F6 and F7.xlsx).

#### Phenotypes scored in the JI0281 × Caméor RIL population

As the JI0281 × Caméor population derives from a wide cross, many phenotypic differences segregate in this population. The purpose of preserving the RIL population is so that these can be studied in detail and for quantitative characters these can be measured with replication. Several discrete characters segregate which are relatively easily scored and mapped.

Among these, segregation of the Mendelian character flower colour occurs, where the recessive white flower colour in Caméor is a consequence of a mutation in the gene *A* encoding a bHLH transcription factor^15^ located in the Caméor v1a assembly at chr6LG2:68330158..68340923. Scoring the phenotype in the F8 generation places the gene in a 0.6 cM interval between markers AX-183569025 (41.42 cM, chr6LG2:68022112 and AX-183572836 (42.12 cM, chr6LG2:69966265) of the F6 and F7 combined genetic map, with 21 co-segregating markers. Mapping in the F7 genetic map narrows the interval to 0.47 cM between AX-183569025 (41.42 cM, chr6LG2:68022112) and AX-183882050 (41.89 cM, chr6LG2:68889090); see Supplementary file 281xCameor F6 and F7.xlsx.

Flower colour is variable among these RILs, with the closely related RILs 575 and 576 having noticeably pink flowers, similar in colour to a *b* mutant^16^. RILs with very pale flowers must have a functional bHLH, but they may easily be mis-scored as white. Conversely, RILs scored as pale coloured flower lines that carry the defective allele are most likely mis-identified F8 individuals.

The difference between tall vs short peas is a Mendelian character corresponding to the gene *Le*^17,18^ on linkage group III, which encodes a gibberellin 3β-hydroxylase. The structural gene is located at chr5LG3:567364535..567368783 on the Caméor v1a assembly. Genetically, this trait is located on the F7 map between the markers AX-183578994 (146.04 cM, chr5LG3:566953568) and AX-183869164 (146.31 cM, chr5LG3:569269414). Again, the scores of three of RILs are inconsistent between genotype and stem length phenotype; two of these (RIL # 196 and RIL # 518) are also inconsistent between their allelic score for *A* and their flower colour.

The seeds of JI0281 are characterised by the presence of small hard bumps on the exterior surface of the seed coat giving the testa a sandpaper-like texture which causes the seeds to make a distinctive gritty noise when rubbed together. The dominant JI0281 allele of a single gene, *Gritty* (*Gty*), on linkage group VI confers the gritty phenotype^19^; however, the molecular basis of this phenotype is unknown. On the F7 map *Gty* cosegregates with AX-183638780 (60.64 cM, scaffold02056:37077) between the adjacent markers AX-183573599 (60.29 cM, chr1LG6:234962989) and AX-183870884 (60.79 cM, super-scaffold6451:261354). The discontinuity in the Caméor v1a assembly^5^, as indicated by scaffold positions of markers, does not occur in the ZW6 assembly^14^. The markers AX-183638780 at ZW6Chr1:313573952, AX-183573599 at ZW6Chr1:312457507, and AX-183870884 ZW6Chr1:314596985 delimit a region of ca. 2 Mb of the ZW6 assembly within which the *Gty* gene lies.

**Figure 4:**
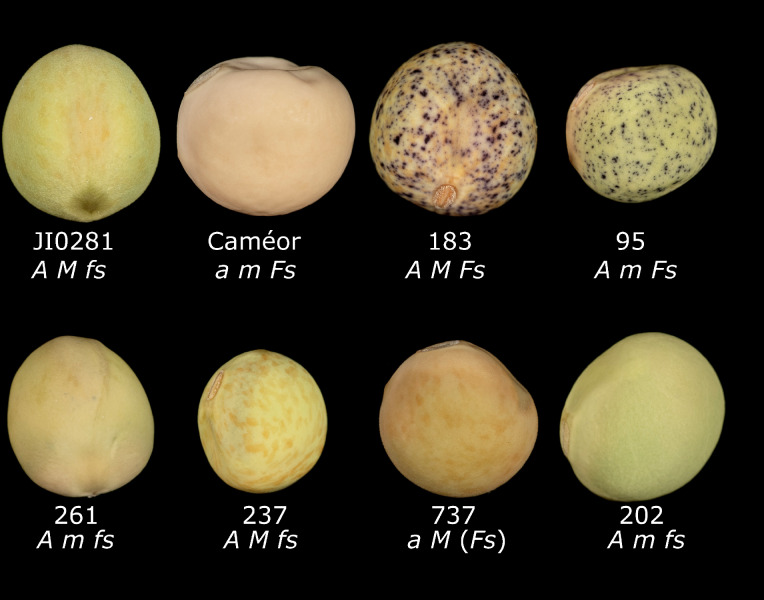
The appearance of seed coats in the JI0281 × Caméor RIL population. Parental lines JI0281, Caméor and F8 RIL seeds are identified by the number below each seed, with the inferred genotype as indicated. For RIL 737 the *Fs* phenotype (bracketed) cannot be seen but it has been inferred from the marker genotype. JI0281 shows brown marbling on a green background with occasional violet spots on the inner surface of the testa. A small number of violet spots can also be seen on the inner surface of RIL 261, but not in RIL202. Caméor has the typical seed coat of a white flowered cultivar. RILs 237 and 737 show that *M* can be scored regardless of the *A* genotype. The seeds shown are approximately 5 mm in diameter.

The RILs were scored for two other dominant seed characters segregating in the JI0281 × Caméor population; the presence of violet spots on the testa (corresponding to either *F* or *Fs*, *Violaceopunctata*,^20^) and the presence of brown marbling (Mendel^21^, corresponding to *M, Marmoreus*^20^) (Fig. 4, Supplementary Fig. 3). The violet anthocyanin pigment spotting is not seen in white-flowered *aa* genotypes, whereas brown marbling is seen in *aa* genotypes, although it can be quite faint. The few violet spots restricted to the inner surface of the testa are a feature of the *fs* allele segregating in this cross. Mapping the violet spotted testa colour on the F7 genetic map indicated that it corresponded to *Fs*, rather than *F* (see below). The intense violet spotting comes from the *Fs* allele of the Caméor parent where it is masked by the *aa* genotype.

The genetic map position of each marker was plotted with respect to violet spotting in Supplementary Fig. 3. The minimum value observed on the plot at AX-183576505 on LG V (105.53 cM, chr3LG5:423158025) corresponds to the position of the major determinant of this character. This confirms that the gene is *Fs* on LG V, rather than *F*, which is on LG III close to *St*^20^. AX-183576505 is located within a gene annotated as SPX domain (Psat3g199720 [SPX = Suppressor of Yeast gpa1, yeast Phosphatase 81 (Pho81), and the human Xenotropic and Polytropic Retrovirus receptor]) and adjacent to a gene annotated as encoding a Myb-like DNA-binding domain (Psat3g199760). Thus, Psat3g199760, which belongs to the anthocyanin regulatory clade of R2R3 *Myb* genes^9^ is a plausible candidate for *Fs*.

#### Marmoreus

Marbling, conferred by *M,* was very difficult to score reliably, with reversals of score for repeated attempts. *M* in *aa* genotypes can be very pale and difficult to distinguish from the background colour of the seed (Supplementary Fig. 3) and the genotypes *FsFs MM* and *FsFs mm* can be difficult to distinguish because the afterimage of the violet spots appears brown on some backgrounds. Therefore, scores for *M* were accepted only when three successive attempts gave the same score, which resulted in 22% missing data. Nevertheless, *M* mapped to an expected position on LG III^20^, with the minimum plot value defined by the marker AX-183878621 (Supp. Fig. 3B). This marker lies adjacent to Psat5g044440, annotated as Uroporphyrinogen-III synthase HemD in the Caméor v1a assembly. This enzyme catalyses the ring closure in heme biosynthesis (https://www.uniprot.org/uniprotkb/G9A2G0/entry) and the accumulation of precursors in this pathway can lead to brown colouration, for example in chicken eggs^22^. It is possible that Psat5g044440 corresponds to *M*, and that patchy expression of heme biosynthesis explains the marbled brown colouration of the testa.

#### Quantitative traits

**Figure 5:**
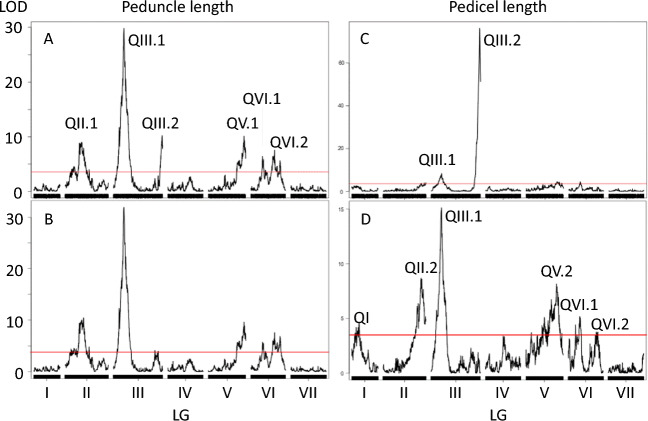
QTL analysis of inflorescence length. QTL mapping was performed using the R/qtl package. Red line represents the LOD threshold of 3.6 at α = 0.05 estimated based on Bayesian credible interval. (**A**) Peduncle length without covariate; (**B**) Peduncle length with *Le* as a covariate, (**C**) Pedicel length without covariate; (**D**) Pedicel length with *Le* as a covariate.

We scored internode length, the length of the peduncle (I2) and pedicel (F) in the F8 generation. Internode length was clearly associated with a region of chromosome 5 LGIII close to *Le*. Caméor carries the R229T amino acid substitution characteristic of *le*; it has the wild type S119 allele of the *Lh* gene nearby so we ascribe the difference in internode length (and plant height) to the Mendelian locus *Le*^17,18^. Here we undertook a QTL analysis which revealed six QTL (Fig. 5) one of which (QIII.2) corresponded to *Le.*

Adjacent to QIII.2 there is a QTL which is just over the significance threshold when the effect of *Le* is taken into account (Fig. 5B). This has the opposite effect to *Le* in *LeLe*, but not *lele* genotypes and may cause a reduction in the significance and magnitude of effect associated with *Le*. An additional QTL on LGIII (QIII.1) had the strongest support and the largest phenotypic effect.

Peduncle and pedicel length were significantly correlated (*r* = 0.53) and several of the QTL peaks overlap (Table 2) suggesting that these may be pleiotropic effects of the same allelic variants.

**Table 2:** Inflorescence length QTL.

QTL	LG	Range (cM)		Peak AX-Marker	cM	LOD	R^2^ effect	Additive	Genomic location Caméor v1a	
Peduncle										
QII.1	II	67.3	87.9	183893027	87	10.5	7.53	6.75	chr6LG2	176959322
QIII.1	III	48.3	50.7	183606332	50	32	25.35	− 12.21	chr5LG3	67580904
QIII.2	III	229.5	233	183578995	232	11.5	8.36	− 7.07	chr5LG3	567367193
QV.1	V	166.7	176.1	183878054	170	9.7	10.14	7.76	chr3LG5	437469581
QVI.2	VI	54.8	144.2	183575601	113	7.6	6.25	6.1	chr1LG6	306151076
Pedicel										
QI	I	13.6	46	183564292	34	4.6	1.93	0.38	chr2LG1	67373343
QII.2	II	178.4	192	183893398	182	8.7	3.28	0.51	chr6LG2	444718856
QIII.1	III	46.6	50.7	183864778	50	15.1	7.3	− 0.75	chr5LG3	74492127
QIII.2	III	231.7	232	183578995	232	76.1	52.31	− 2.03	chr5LG3	567367193
QV.2	V	121.8	156.9	183873583	147	8.2	3.98	0.56	chr3LG5	359645629
QVI.1	VI	33.6	141.3	183584457	55	5.2	2.93	0.47	chr1LG6	100332386

Details of the position and range of the QTL indicated in Fig. 5 are shown. The QTL confidence interval estimated based on the Bayesian credible interval define the cM Range (these are positions on the F7 map which are generally larger than for the F6 map, see Supplementary Fig. 2). The genomic locations of the QTL peaks on published assemblies are given (Caméor v1a^5^, ZW6^14^, ZW6 positions are indicated on the Excel file version of this table).

### JI2202 × JI2822

Both *P. sativum* and *P. abyssinicum* occur in Ethiopia and they are distinct both in phenotype and their use^23^. The names given to pea taxa have been much debated, but it is clear that *Pisum* as a taxon in embedded within *Lathyrus*, which in turn is embedded within *Vicia*^24^. Within *Pisum* there is considerable differentiation of sub-taxa^6,7,25–27^ but these taxa have equivocal relationships to one another and their taxonomic level (species, subspecies or variety) has been discussed at length such that *P. abyssinicum* is sometimes designated as *P. sativum* ssp *abyssinicum* or even *Lathyrus schaeferi*^28^. Nevertheless, *P. abyssinicum* is a well separated and relatively homogeneous group within *Pisum* and is a source of novel disease resistance alleles^29,30^.

JI2822 is a *P. sativum* accession derived from the cross between JI0015 and JI0399 which has been used for the generation of a Fast Neutron deletion population^31^. It was considered that this cross would provide many markers in relation to JI2822 and could shed light on the domestication of *P. abyssinicum*^32^.

JI2202 is a *P. abyssinicum* accession, designated ‘*P. sativum abyssinicum* landrace DCG0563’^5^, while JI2822 is a *P. sativum* genetic stock line. The F2 population derived from the cross JI2202 × JI2822 comprised 199 individuals which were selfed for thirteen generations. Populations generated after F2 suffered from severe attrition due to the failure of RILs to set seed such that only 117 lines survived at F13. Such infertility has been noted previously for this inter-subspecific cross^28,33^. Structural differences between the *P. sativum* and *P. abyssinicum* genomes have been described^28,34^, which might explain the attrition of progeny lines.

#### The genetic map of JI2202 × JI2822

**Figure 6:**
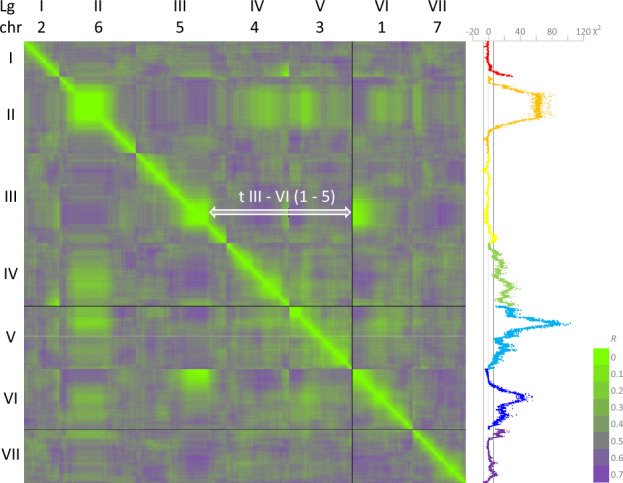
A heat map of pairwise marker distances in the JI2202 × JI2822 RILs. The colour coding of the heatmap (green to purple) is shown on the bottom right. The values for *R*, the fraction of recombinant inbred lines that have recombined the parental alleles, are shown for every 10th marker. A double-headed arrow indicates a translocation between LG III (chr5) and LG VI (chr1). On the right the χ^2^ value for the segregation ratio is plotted for all markers, where the expectation is a 1:1 segregation. The sign of the χ^2^ is positive for an excess of JI 2202 alleles and negative for an excess of JI 2822 alleles. The parallel lines at the base of the plot correspond to the values χ^2^ − 6.635, 0 and 6.635, representing the 1% confidence interval.

A total of 15,011 markers were positioned on the JI2202xJI2822 genetic map (Supplementary files JI2022xJI2822.xlsx and JI2202xJI2822.html). This map has several unusual features, as illustrated by a heat map of the genetic distances between successive markers (Fig. 6.)

The green-shaded diagonal in Fig. 6 represents closely adjacent markers. Remarkably, Fig. 6 also depicts very low *R* values for blocks of markers assigned to different linkage groups (green-shaded areas off the diagonal). These usually correspond to regions of strong segregation distortion as seen in the χ^2^ plot on the right, most obviously for the central part of LG II reflected in regions of LGs IV, V, VI and VII that similarly show segregation distortion in favour of *P. abyssinicum* alleles. The low crossover rate in this part of LG II can be seen clearly in a comparison of the genetic maps of JI0281 × Caméor and JI2202 × JI2822 (Supplementary Fig. 4). This probably corresponds to a structural rearrangement and would be consistent with an inversion. A pericentric inversion of chromosome 6 (LG II) was proposed to distinguish *P. abyssinicum* from *P. sativum*^34^.

For LG III (chromosome 5) and LG VI (chromosome 1), a substantial number of markers close to the end of LG VI show no recombination with respect to markers centrally placed on LG III. This is seen clearly in the Threadmapper analysis (Supplementary Fig. 5) where the two linkage groups are connected. Markers with identical scores on the two chromosomes are from chr5LG3:301400471..468433969 to chr1LG6:752056..31775089 (ZW6Chr5:362651675..532607597, Chr1:1104220..34978714), suggesting that a translocation breakpoint occurs within this region.

A chromosome 1–5 translocation was proposed to distinguish *P. sativum sativum* from other pea taxa^5^, and both parents of JI2822 have the standard *P. s. sativum* karyotype^35^. Thus, *P. abyssinicum* retains the ancestral karyotype with respect to these two chromosomes, consistent with the proposal that *P. abyssinicum* has been domesticated independently of *P. s. sativum*^6,7,27,33^.

Another translocation may be affecting LG I and LG IV (Fig. 6). However, this interpretation is confounded by segregation distortion at the corresponding locations in both linkage groups. In contrast to the LG III – LG VI translocation discussed above, there were no pairwise marker associations for which there were zero recombination events. In the Threadmapper display (Supplementary file LG1-IV-JI2202-JI2822.html) this putative translocation appears as an association of chromosome ends.

#### Phenotypes scored in the JI2202 × JI2822 RIL population

**Figure 7:**
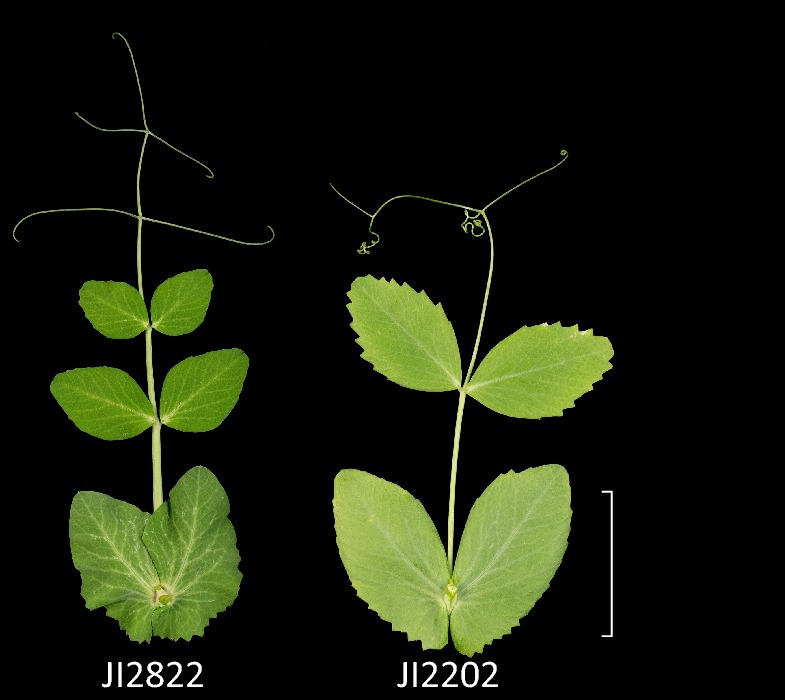
Leaves of JI2822 and JI2202. The JI2202 leaf has the typical grey-green colour of *P. abyssinicum*, strong serration of the leaflet and stipule base, lack of axil ring pigmentation and one pair of leaflets. Leaves are from node 10 of 4-week-old plants. Three plants each of JI2202 and JI2822 were planted on the 21st and 20th September 2023 respectively and grown in the summer regime, i.e. without supplementary heating or lighting. All three JI2202 plants flowered at node 14 and no leaves had more than two leaflets. All three JI2822 plants flowered at node 8 and changed from two leaflets per node to 4 leaflets per node at node 10. The first two leaves of both genotypes are scale leaves, lacking leaflets. The scale bar is 4 cm.

Despite extensive segregation distortion in this cross, several phenotypic differences segregated in a way that was straightforward to interpret and score. The absence of axil ring pigmentation is a feature of *P. abyssinicum*^20^, Fig. 7, and this trait was found to map between AX-183564315 (chr2LG1:80470984) and AX-183581090 (chr2LG1:70183395). This region of the genome contains six genes annotated as encoding Myb transcription factors (Psat2g042040 = PsMYB15, Psat2g042600, Psat2g043400, Psat2g043680 = PsMYB16, Psat2g047760 = PsMYB17, Psat2g049080) in the Caméor v1a assembly^5^ and which have corresponding PsMYB numbers^9^.

*P. abyssinicum* is characterised by having a maximum of one pair of leaflets per leaf (*up*; *unipetiolle*)^20,23^, in contrast to *P. sativum* where two or more leaflet pairs is common. Accordingly we scored the maximum number of leaflet pairs in these RILs. The difference in leaflet number between RILs with the *P. abyssinicum* allele vs the *P. sativum* allele occurs between AX-183594307 (chr3LG5:91712588) and AX-183866382 (chr3LG5:98802650) suggesting the location of *up* is in the middle of LG V (Supplementary Fig. 6).

Leaflet serration is another characteristic of *P. abyssinicum*, reportedly due to a gene *Serratus* (*Ser*)^20^, where the dominant allele confers serration. When leaflet margins were scored as serrated or entire (including mildly dentate) in the JI2202 × JI2822 RILs, this difference in phenotype was associated with two different genomic locations on the genetic map (Supplementary Fig. 6), one on LG III, the other on LG VI. In both cases, it is the *P. abyssinicum* allele that confers serration or dentation. The gene *Td* (*scalaris forma*) confers dentation in *P. sativum*^20^ and was mapped about 2/3^rds^ of the way along LG III^36^, in approximately the same location as *Ser1* in this RIL population. Note that *Ser1* and *Ser2* coincide with the position of the translocation distinguishing *P. sativum* and *P. abyssinicum*, suggesting that the appearance of two peaks is an artefact of the way the genetic map is drawn.

## Discussion

The RIL populations and maps presented here capture diversity within domesticated field pea (JI0281 × Caméor) and between two independently domesticated pea types (JI2202 × JI2822). The former cross provides a map resolution of approximately 0.1 cM, which is often sufficient to define a small number of genes that co-segregate within a particular interval. The latter cross provides resolution of 0.25 cM.

### Structural features

The genetic map of the JI0281 × Caméor cross is largely collinear with the Caméor v1a assembly (Fig. 3) but with some large scale non-linearities and many scattered points of non-correspondence, similar to those revealed in the comparison of this assembly to earlier genetic maps^5^. Comparison of the JI0281 × Caméor genetic map to the more recent ZW6 genome assembly^14^ shows a much simpler correspondence, with minor deviations from collinearity, for example at the ends of LG II (chromosome 6) and LG VII (chromosome 7). This improvement in the agreement between genome assembly and genetic mapping is to be expected from the increased read lengths of the ZW6 assembly that span the abundant Ogre elements in the pea genome^5,37^.

In contrast with the simple structure of the JI0281 × Caméor genetic map, the JI2202 × JI2822 genetic map has two obvious atypical features. The first is the translocation between LG III and LG VI (chromosomes 5 and 1, respectively) and the second is extensive segregation distortion (Fig. 6). Conicella and Errico^34^ also reported a translocation and a pericentric inversion that distinguished *P. sativum* and *P. abyssinicum;* their pericentric inversion distinguishing *P. sativum* and *P. abyssinicum* could correspond to the region of depressed recombination rate observed in LG II of the JI2202 × JI2822 RIL population (Supplementary Fig. 4). In Conicella and Errico’s crosses with translocation lines from^38^, the evidence for a translocation involving LG I, chromosome 2 (in the current naming system) is strong and that would be consistent with the association seen between LG I and LG IV (Fig. 6).

These considerations are rather tentative because the correspondences in *Pisum* chromosome designations are uncertain and the relationship between JI2202 and the *P. abyssinicum* accession used by^34^ is not clear. It is relevant that more than one *P. abyssinicum* karyotype was reported by these authors, which seems at variance from currently accepted views of the narrow diversity within that taxon. Furthermore, the LG III–VI translocation described here is in agreement with Kreplak et al.^5^, i.e. that a chromosome 1–5 translocation is derived within *P. sativum*.

### Phenotypes

The JI0281 × Caméor map positions *Gty* within a 2 Mb region of the chromosome 6 LG II ZW6 assembly^14^ that includes many candidate genes. However, the Caméor allele is *gty* and therefore the gene may be defective, or absent from the Cameor v1a assembly, and therefore difficult to identify. Refining the candidate list of genes for *Gty* awaits a sequence assembly of this region from JI0281, highlighting the need for chromosome level assemblies of pangenomic data.

The *Fs* gene was associated with Psat3g199760 encoding a Myb transcription factor. Psat3g199760 is also known as *PsMYB37* in JI2822 and it appears to contribute to the coloration of the lateral petals^9^. *PsMYB37* expression was not detected in young seeds^9,39^ probably because immature pea seeds do not exhibit the *Fs* type pigmentation and the seeds were of cv. Caméor which lacks this pigmentation because it is white flowered (see Fig. 4).. If this transcription factor is also involved in seed coat anthocyanin production then it would need to be proposed that this transcription factor acts differently in flowers and seeds as *Fs* and *fs* genotypes do not have obvious differences in flower colour.

The gene *M* was difficult to score, but a position on the map was obtained which suggested that variation in a Uroporphyrinogen-III synthase may be the cause of the accumulation of the brown pigment in the *MM* genotypes. Further assessment of this candidate awaits additional genetic, genomic and transcriptomic analyses.

The quantitative traits peduncle and pedicel length were investigated; these are stems corresponding to the I2 and F meristems^40^ and therefore may be expected to be regulated differently from the main stem (I1). However, it was clear that *Le* vs *le* has a major affect on these three different structures (Fig. 5, Table 2). Additional loci were identified that regulate the length of either or both of these structures, with a highly significant QTL of large effect at the opposite end of LGIII from *Le*. In his 1866 paper Mendel reported “An experiment on flower stems of different lengths gave on the whole a rather satisfactory result, although distinction and classification of the forms could not be accomplished with the certainty that is indispensable for correct experiments”^21,41^. It is possible that this major QTL would have been what Mendel observed and it would have been particularly striking in crosses in which internode length did not segregate. The additional QTL, behaving as modifiers, may explain the comment on the uncertainty of their serial arrangement.

The genetic map derived from the cross between *P. sativum* and *P. abyssinicum* has lower resolution than that derived from JI0281 × Caméor because the RIL population is smaller and the data is biased by extensive segregation distortion. Despite these difficulties, candidate genes for three major traits distinguishing these taxa were identified. In conjunction with other approaches, these candidates can be investigated further.

Lack of axil ring pigmentation is a characteristic of *P. abyssinicum* accessions; a phenotype conferred by the recessive *d* allele on LG I^20^. This trait mapped to the expected position on LG I of the JI2202 × JI2822 genetic map, within a 10 Mb region rich in R2R3 *Myb* genes, five of which are known to be expressed in pea flowers^9^. These genes are also candidates for the regulation of anthocyanin pigmentation in axil rings.

In *P. abyssinicum* leaflet number does not exceed a single pair^20,23^. This trait was mapped to a single locus on LG V of the JI2202 × JI2822 genetic map, corresponding to a 7 Mb segment of chromosome 3. In contrast, leaflet serration, which is another characteristic of *P. abyssinicum* (Fig. 7^23^) and thought to be determined by a single gene *Ser*^20^, appeared to be resolved into two genetic loci, which we have designated *Ser1* and *Ser2. Ser1* and *Ser2* correspond in position to the translocation point distinguishing the karyotypes of JI2202 and JI2822 and appear as two loci because the genetic map is presented in a linear form rather than as the branched structure shown in the accompanying file LG1-IV-JI2202-JI2822.htm. There is much confusion in the literature about the relationship between *Ser* and *Td*^36,42–44^. *Td* determines weak dentation in *P. sativum* while serration in *P. abyssinicum* is assigned to *Ser*^20^. The differences in leaf serration observed in this study may correspond to allelic variation at these two loci, but in the JI2202 × JI2822 RIL population increased serration is associated with the *P. abyssinicum* allele at these two loci. For this reason, it seems unwise to assert that *Ser1* and *Ser2* correspond to *Td* and *Ser* respectively. The possibility exists that *Ser* and *Td* are in fact the same genes^44^. We note that, in the Caméor v1a assembly, the marker AX-183567759 associated with *Ser1*, is 70 kb from the gene Psat5g237280 which has sequence similarity to *JAGGED* in *Arabidopsis thaliana*^45,46^.

## Conclusions

We have investigated genomic and phenotypic differences segregating in two RIL populations representing very different phenotypic groups within *Pisum*. These populations, together with their associated marker data, are available from the John Innes Pisum Germplasm collection.

## Methods

### Plant materials

Lines designated JIxxxx were initially obtained from the John Innes *Pisum* germplasm collection and grown singly in pots in a glasshouse. In spring, autumn and winter the greenhouse was heated to 18 °C by day and 12 °C at night and supplementary lighting (Heliospectra LED Elixia lamps) was used to maintain a 16 h photoperiod. In summer, neither heating, nor lighting was provided. The plastic pots were 9 cm diameter containing a Peat/Loam/Grit mix (65:25:10) supplemented with 3 kg/m3 Dolomitic limestone; these were placed on capillary matting which was soaked twice a day by automated watering. Plants were supported by canes. The F9 of JI281xCaméor and the F13 of JI2202xJI2822 were grown in the winter of 2022–2023. The John Innes *Pisum* germplasm collection has the UK national collection of *Pisum* and operates within the International Treaty for Plant Genetic Resources for Food and Agriculture (ITPGRFA) and the collection is registered with the ITPGRFA Multilateral System. Seed obtained from this source are obtained and distributed under the terms and conditions of the Standard Material Transfer Agreement of the ITPGRFA. Information on these plant phenotypes and the original source of the material is available at the website of the John Innes Pisum Germplasm collection. In addition useful information about pea genes can be found at the website of the Polish Genebank (http://pw.ihar.edu.pl/wp-content/uploads/2020/11/The-Catalogue-of-Pisum-genes_02.01.2019_small.pdf). Recombinant inbred lines were obtained by single seed descent from an F_2_ population. Thus the material obtained for this study is compliant with the ITPGRFA and the material generated within this study will be distributed in the same way. Thus all plant materials comply with relevant institutional, national, and international guidelines and legislation.

DNA preparation was from leaf samples using Qiagen (ThermoFisher) or oKtopure (Biosearch Technologies) kits as described by the manufacturers.

### The Axiom array

In order to build a genotyping array for pea, Single Nucleotide Polymorphism (SNP) identified in three different projects were used:

- 15,000 SNPs used for the design of the Genopea 13.2 K array^47^;
- 15,000 SNPs identified in breeding panels of the PeaMUST project^48^ using the exome capture technique as described in Aubert et al.^49^;
- 100,000 SNPs selected out of the ca 17.2 M SNPs identified in Kreplak et al.^5^ from resequencing data for 42 *Pisum* accessions. A 10% Minor Allele Frequency additional filter was applied.

Corresponding VCF files were submitted to Thermo Fisher Scientific for probeset design and 84,691 SNPs were successfully tiled on the pea Axiom array. SNPs and their position on the pea genome are listed in Supplementary Table 3.

Genotyping of the F6 population with the array was performed using the Affymetrix GeneTitan platform following the manufacturer’s guidelines. Genotyping of the JI0281 × Caméor F7 RILs and the JI2202 × JI2822 RILs was carried out by Neogen Ltd (Neogen Europe, Ayr, Scotland), whereas the JI0281 × Caméor F6 RILs were genotyped by Gentyane INRAe Genomics platform (https://shorturl.at/zBMZ4,^50^).

Results were analysed with the Axiom Analysis Suite v5.0.1.38 using diploid threshold configurations and default DishQC settings (DQC ≥ 0.82 and call rate > 0.97). Markers classified into the Poly High Resolution category were kept for further analyses of the F6 data, but additional classes were included for the F7 and F13 data as indicated in Supplementary Table 2 and on the map data files.

### Mapping methods

Genetic mapping used the method of^10^ and Threadmapper^11^ but with the principal component analysis based on the scored data rather than the pairwise marker distance matrix. The source code and an example with an input and outputs are available at: https://github.com/threadmapper/ril-maps

## Supplementary Information

Supplementary Information 1.

Supplementary Information 2.

Supplementary Information 3.

Supplementary Information 4.

Supplementary Information 5.

Supplementary Information 6.

Supplementary Information 7.

Supplementary Information 8.

Supplementary Information 9.

Supplementary Information 10.

## Data Availability

Data is available in the Supplementary information, the RILs are available through the JI Genetic Resources Unit (https://www.seedstor.ac.uk/search-panel.php) the Axiom array is available commercially and the sequences of the probes are provided.
