# Supplementary material for: Recombinant inbred lines derived from wide crosses in Pisum
Source: Sci Rep. 2023 Nov 21;13:20408. doi: 10.1038/s41598-023-47329-9 (PMC10663473; doi:10.1038/s41598-023-47329-9)
Supplement: Supplementary file 7 — Supplementary Information 7. [file 41598_2023_47329_MOESM7_ESM.pptx]

## Slide 1
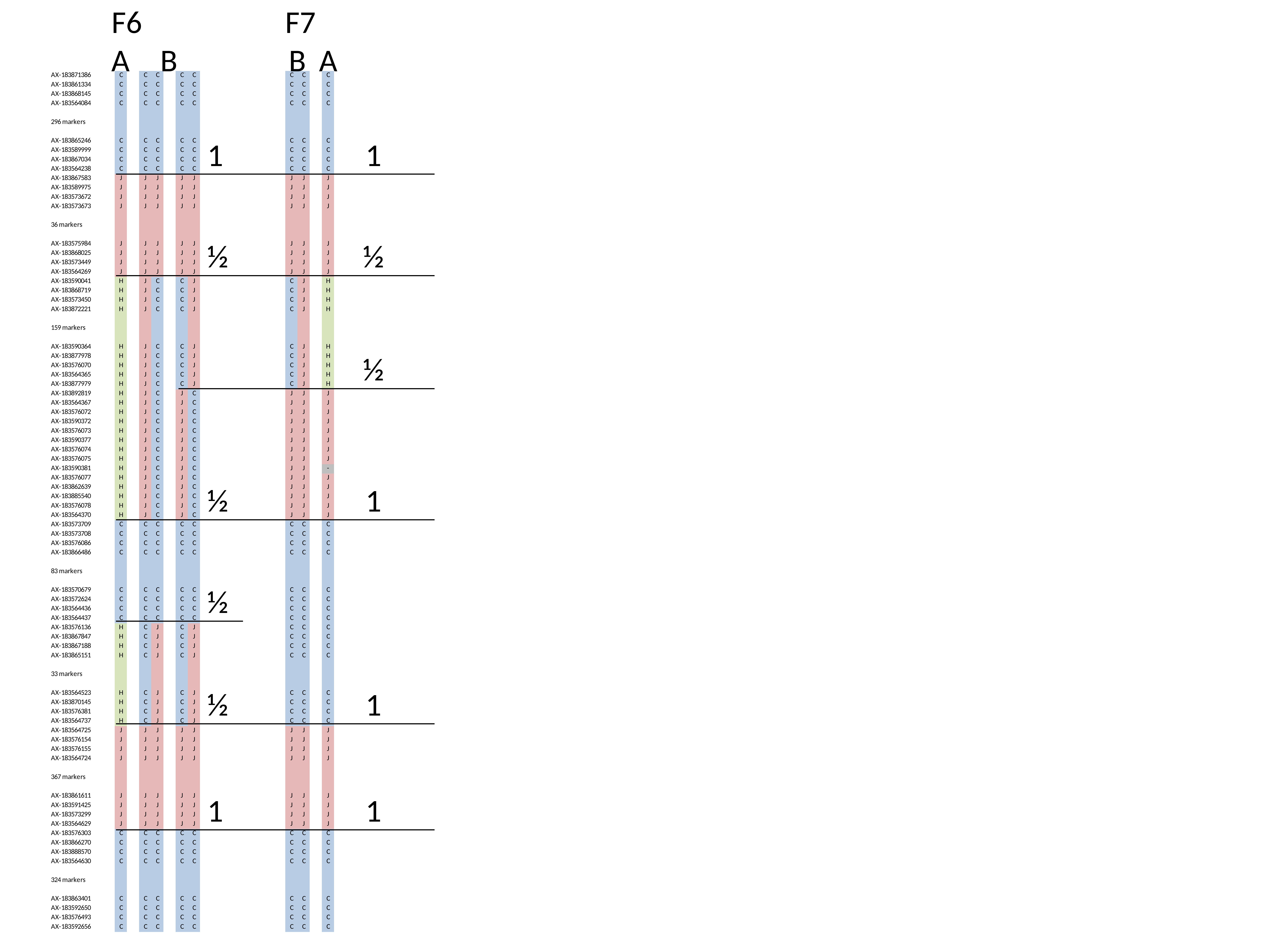

F6
F7
A
B
B
A
1
1
½
½
½
½
1
½
½
1
1
1

## Slide 2
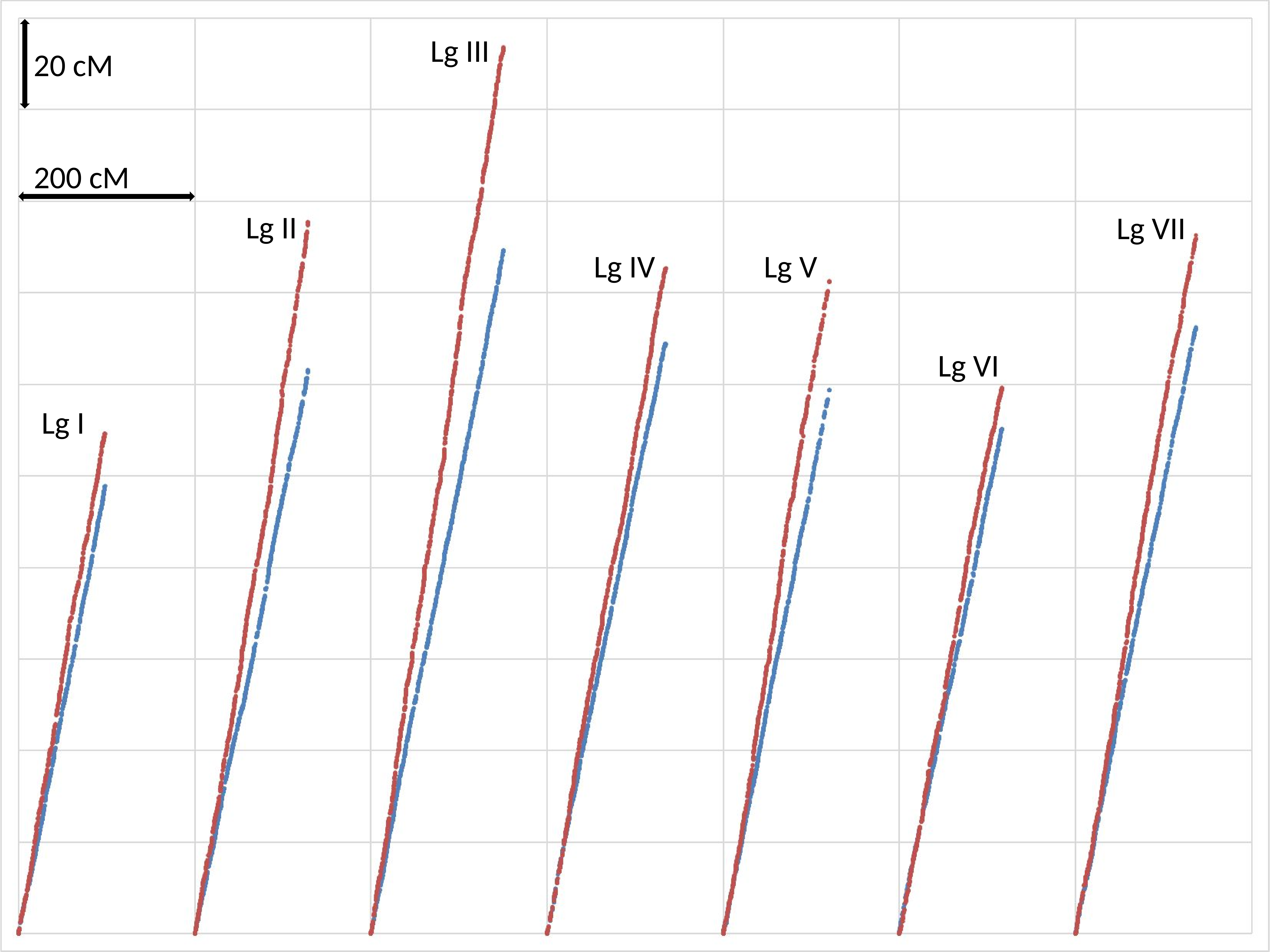

Lg III
20 cM
200 cM
Lg II
Lg VII
Lg IV
Lg V
Lg VI
Lg I

## Slide 3
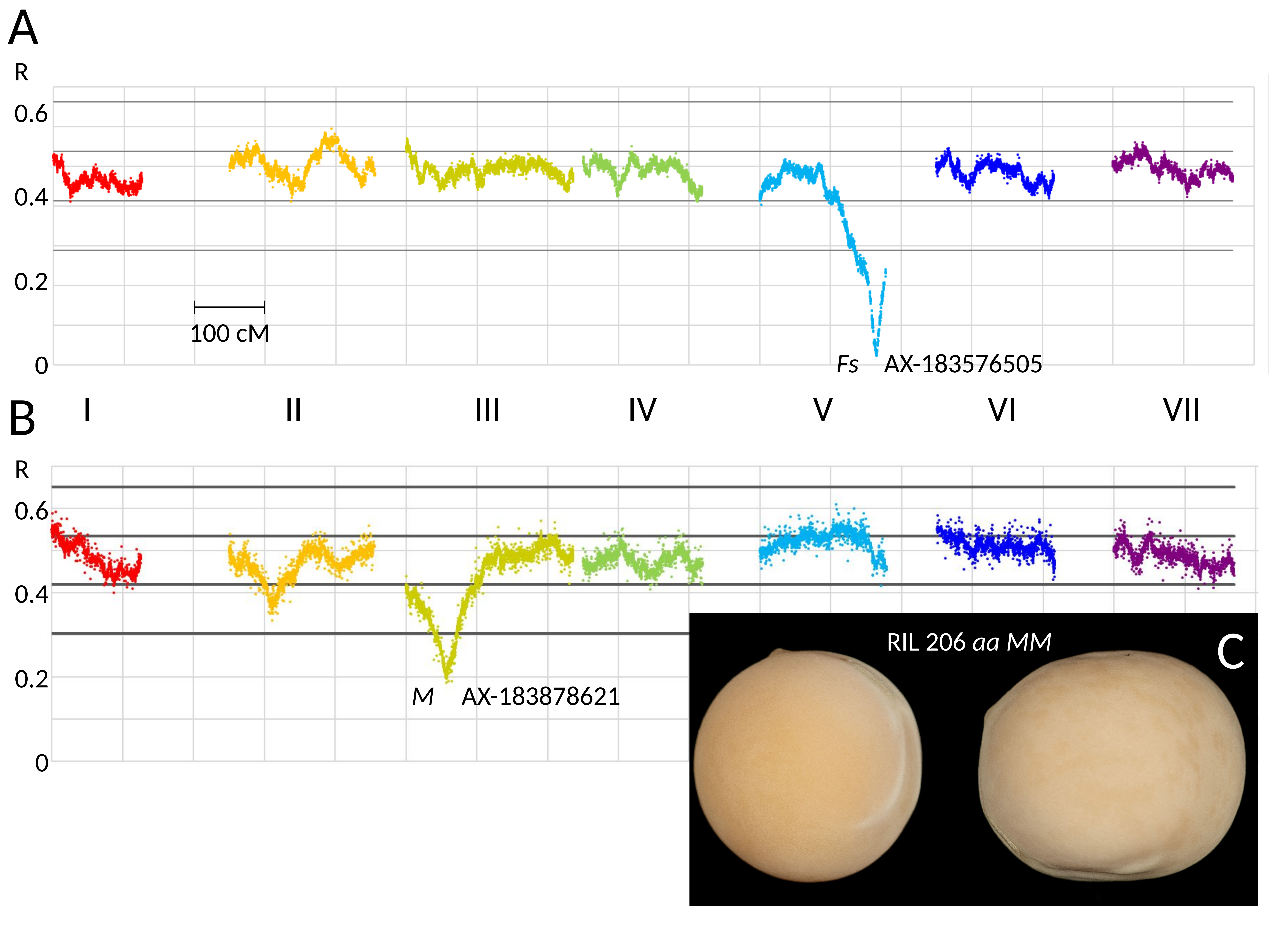

A
R
0.6
0.4
0.2
100 cM
Fs
AX-183576505
0
B
I
II
III
IV
V
VI
VII
R
0.6
0.4
0.2
M
AX-183878621
0
C
RIL 206 aa MM

## Slide 4
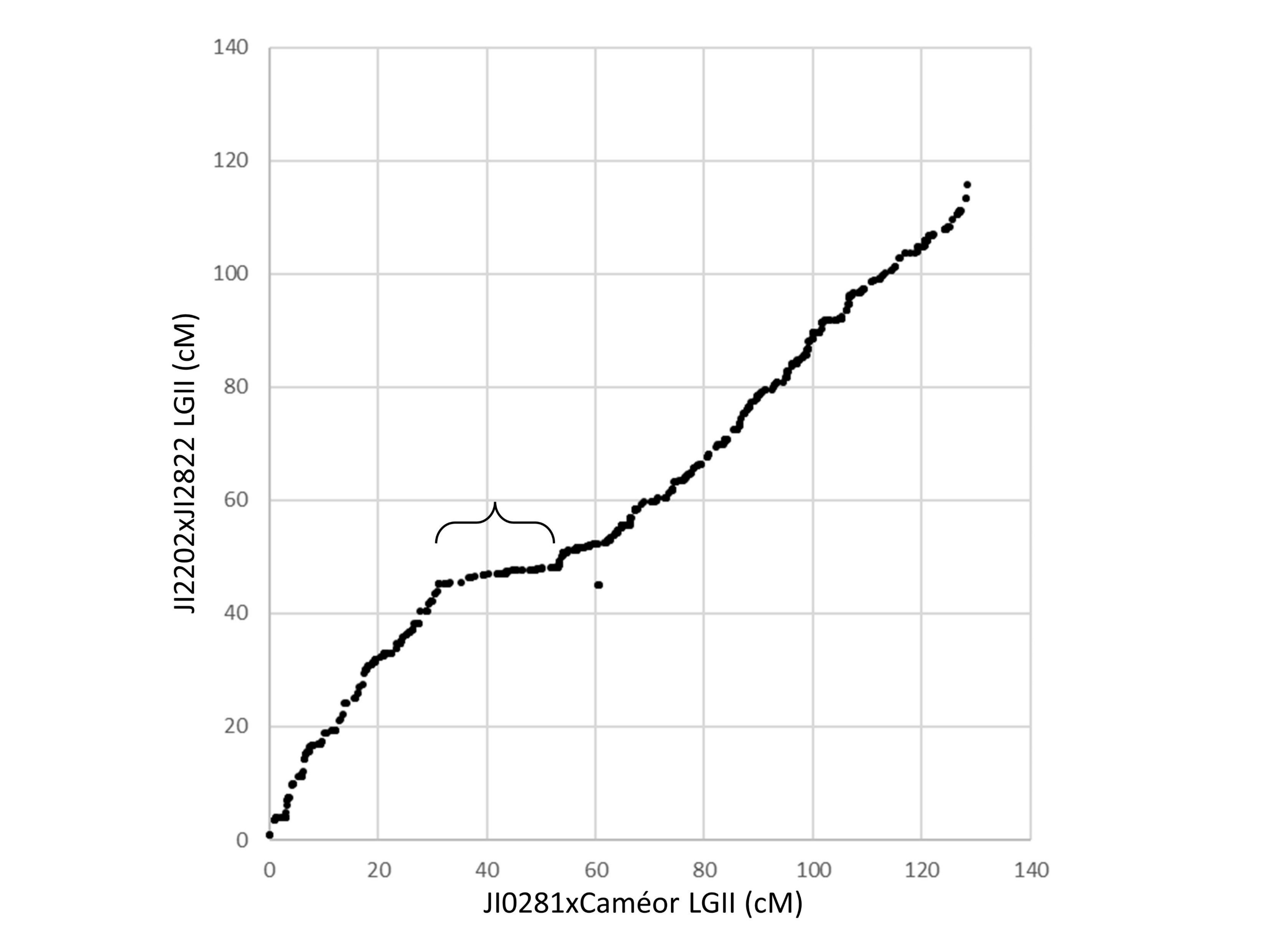

## Slide 5
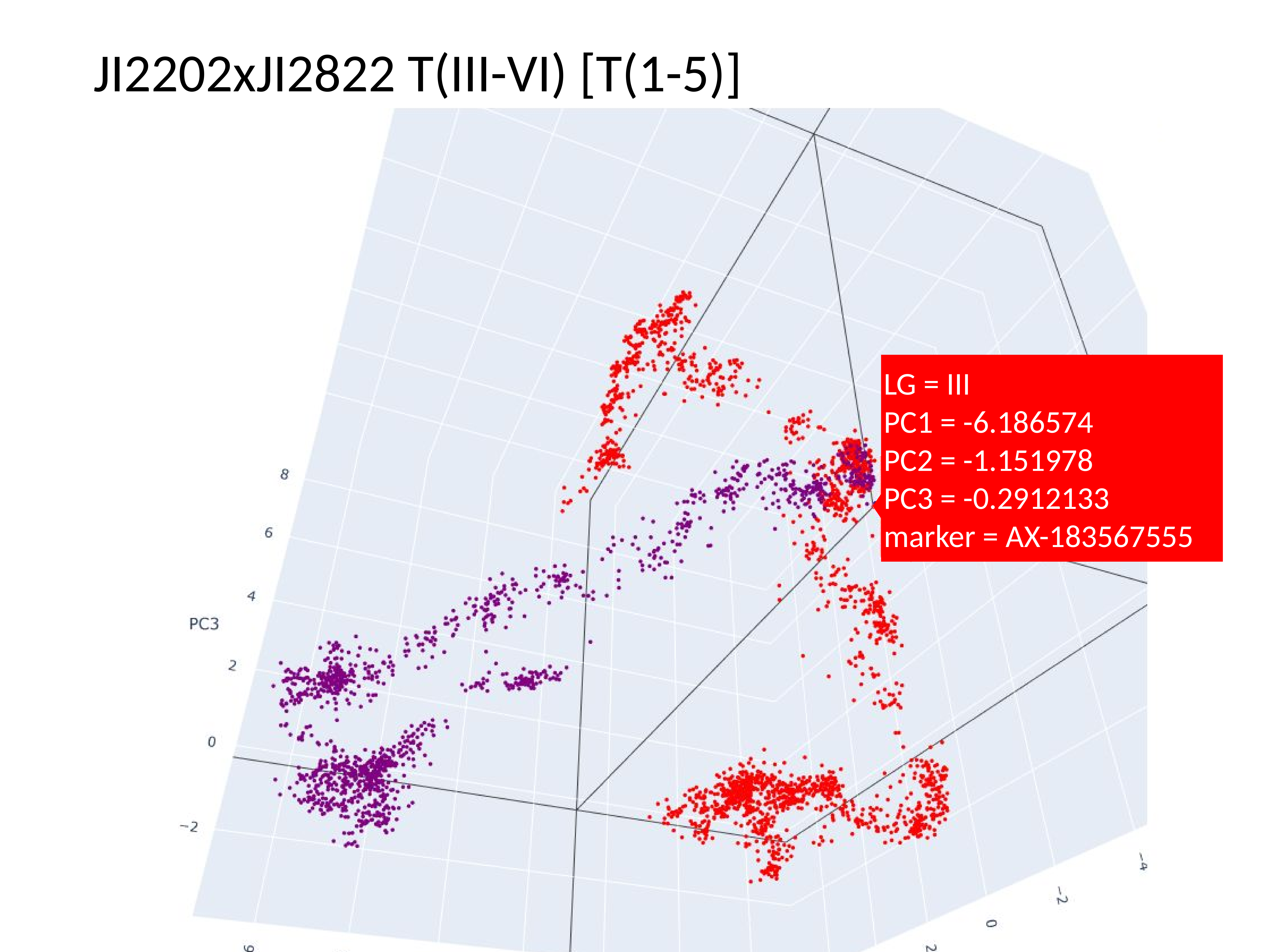

JI2202xJI2822 T(III-VI) [T(1-5)]
LG = III
PC1 = -6.186574
PC2 = -1.151978
PC3 = -0.2912133
marker = AX-183567555

## Slide 6
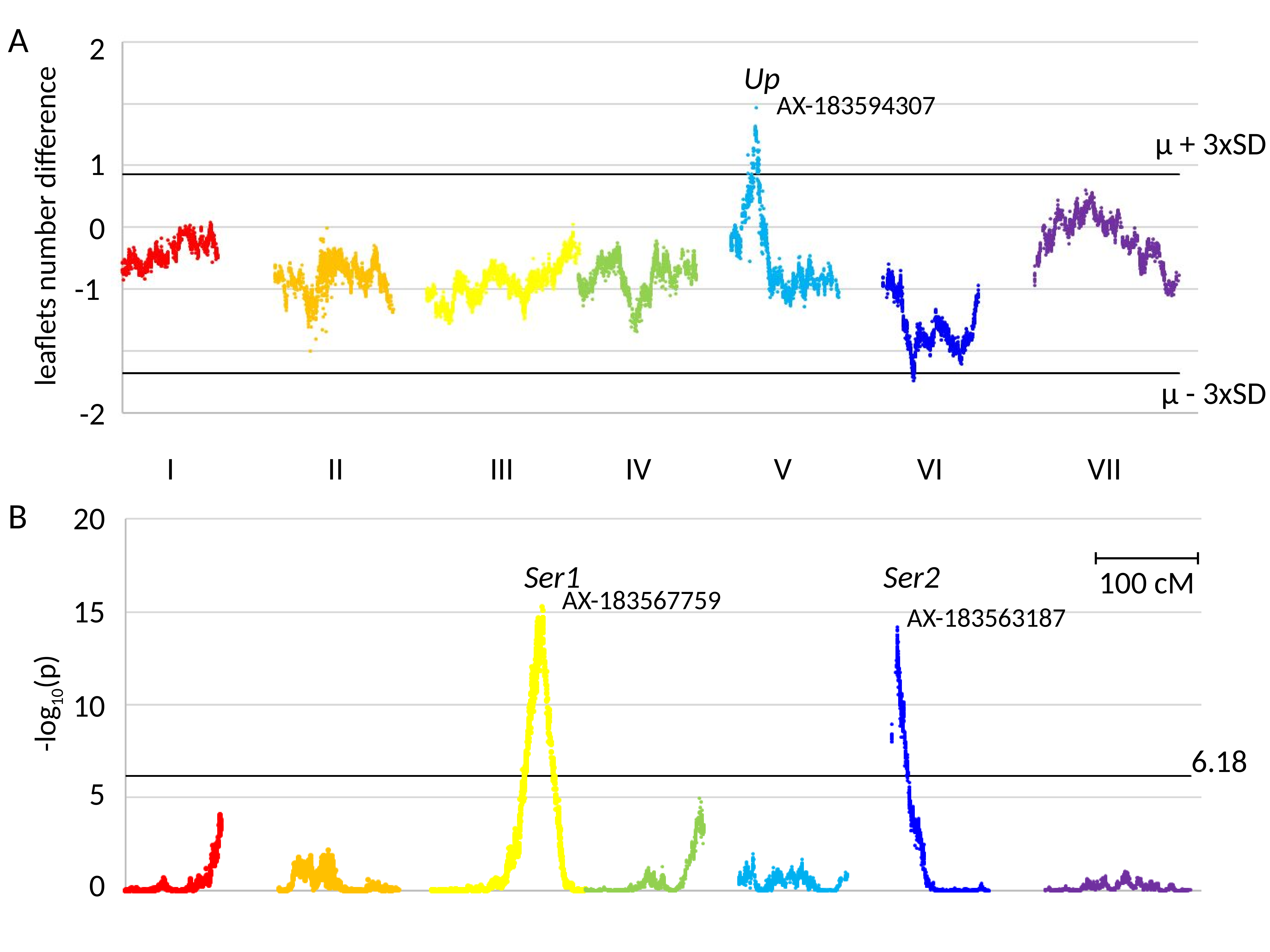

A
2
 Up
AX-183594307
 µ + 3xSD
1
0
leaflets number difference
-1
 µ - 3xSD
-2
I
II
III
IV
V
VI
VII
B
20
100 cM
 Ser1
 Ser2
AX-183567759
15
AX-183563187
10
-log10(p)
 6.18
5
0
